# Unique challenges experienced during the process of implementing mobile health information technology in developing countries

**DOI:** 10.1186/1472-6963-14-S2-P87

**Published:** 2014-07-07

**Authors:** Siobhan O’Connor, John O’Donoghue, Joe Gallagher, Tiwonge Kawonga

**Affiliations:** 1 Health Information Systems Research Centre, Department of Accounting, Finance and Information Systems, University College Cork, Cork, Ireland; 2 Department of Information and Communication Technology, Mzuzu University, Mzuzu, Malawi

## Background

Healthcare in developing countries faces a number of challenges due to economic constraints, poor infrastructure, a shortage of trained clincial staff, extreme climate and geographical barriers among others. Information and communication technologies are seen as one approach to address these issues and improve inequalities in healthcare facing low and middle income countries. In particular mobile Information Technology (IT) is being explored as an underarching framework due to its portability, flexibility, low cost, and widespread network coverage [1].

However implementing mobile IT in this context presents a number of unique barriers. Current research in this domain focuses on barriers of generic IT adoption without considering the specifics of mobile technology. Furthermore, there is a lack of emphasis on the different phases of the implementation process and what barriers emerge during each of these.

## Materials and methods

The objective of this paper is to highlight barriers to mobile IT in resource poor settings during the various stages of implementation based on Cooper and Zmud’s [2] model of Technological Diffusion. The five phases of the model; initiation, adoption, adaptation, acceptance, routinization and infusion, are reviewed and empirical studies of mobile IT in developing countries related to each phase of the model are presented and discussed.

## Results

This study reveals that a number of unique socio-cultural and technological factors, such as mobile technology that is not adapted to culturally ‘fit’ with low resource settings, computer illiteracy, unreliable energy supplies and poor network coverage in rural areas, can hinder mobile IT implementation in developing regions. Moreover, it highlights specific barriers of mobile IT at each stage of the implementation process.

## Conclusions

Mobile IT has the potential to address health inequalities and overcome resource constraints in developing countries. However specific barriers associated with this context need to be considered when implementing mobile technology. Mobile IT needs to be included as part of a larger healthcare strategy for it to be successful and sustainable as this helps overcome some of the barriers to implementation.

## References

[B1] World Health Organisation (WHO) mHealth: New horizons for health through mobile technologies Global Observatory for eHealth series 2011 14 Geneva: WHO

[B2] Cooper RB Zmud RW Information technology implementation research: a technological diffusion approach Management Science 1990 14 123 139 10.1287/mnsc.36.2.123

