# Erratum to: Unique challenges experienced during the process of implementing mobile health information technology in developing countries

**DOI:** 10.1186/s12913-016-1345-2

**Published:** 2016-04-06

**Authors:** Siobhan O’Connor, John O’Donoghue, Joe Gallagher, Yvonne O’Connor, Tiwonge Kawonga

**Affiliations:** School of Nursing, Midwifery & Social Work, University of Manchester, Manchester, UK; Department of Primary Care and Public Health, Global eHealth Unit, Imperial College London, London, UK; Global Health Research Group, University College Dublin, Dublin, Ireland; Department of Accounting, Finance and Information Systems, Health Information Systems Research Centre, University College Cork, Cork, Ireland; Information & Communication Technology Department, Mzuzu University, Mzuzu, Malawi

Upon publication of this Poster Presentation [1] it was found that one of the original authors, Yvonne O’Connor, had accidentally not been mentioned and affiliations were displayed incorrectly. The correct list of authors and their respective affiliations can now be found in this erratum.
